# Diaphyseal Tibial Osteosarcoma With Myiasis

**DOI:** 10.7759/cureus.32718

**Published:** 2022-12-20

**Authors:** Harshika K Saluja, Shivshankar Jadhav

**Affiliations:** 1 Department of Surgery, Jawaharlal Nehru Medical College, Datta Meghe Institute of Medical Sciences, Wardha, IND; 2 Department of Orthopaedic Surgery, Jawaharlal Nehru Medical College, Datta Meghe Institute of Medical Sciences, Wardha, IND

**Keywords:** rare, chemotherapy, tibia, maggots, diaphyseal osteosarcoma

## Abstract

Osteosarcoma is a tumour that can originate in any bone and is the most frequent malignant tumour of the skeleton. It typically develops close to the metaphyseal growth plates in the limbs' long bones. The three most prevalent places are the femur, tibia, and humerus. Additional locations include the pelvis, skull, and jaw. Diaphyseal osteosarcoma involves a smaller population and is highly uncommon. Conventional kinds of osteosarcoma, such as osteoblastic, chondroblastic, and fibroblastic types, as well as telangiectatic, multifocal, parosteal, and periosteal types, are some of the variations of the disease. The primary bone tumour (cancerous) is generated by the formation of immature bone and primarily affects adolescents. We present a case of a 45-year-old menopausal female with left tibial osteosarcoma of the proximal 1/3 diaphysis infected with maggots and complaints of left knee pain and tingling.

## Introduction

A high-grade malignant mesenchymal tumour known as osteosarcoma is distinguished by the tumour cells’ ability to generate immature bone or osteoid. Adolescence and old age are both susceptible times for this tumour to manifest, i.e., it has a bimodal distribution. Osteosarcomas account for 3% of all paediatric cancers in children [1-3]. The incidence peaks during the pubertal growth spurt. In adults, osteosarcoma is typically diagnosed as a secondary tumour. Osteosarcoma can be preceded by Paget’s disease or another malignancy. Adults are susceptible to osteosarcoma for various reasons, including hereditary abnormalities, benign bone cancers, Paget’s disease, and previous radiation or chemotherapy [4]. Osteosarcoma patients typically complain about swelling, restricted joints, and other movements; physical findings include palpable masses and localised pain. Here, myiasis has been discussed in conjunction with diaphyseal osteosarcoma of the tibia. The phrase derived from the Greek word "myia", which means fly, was first used by F.W. Hope in 1940. Laurence gave the first account of it in 1909. Zumpt has characterised it as the infection of living people and vertebrate animals by dipterous larvae that feed for a while on either living or dead host tissue, liquid body material, or swallowed food [5]. An uncommon disorder called human myiasis causes infestations that can harm organs or tissues vulnerable to egg laying, larval growth, and necrotic lesions that serve as an excellent substrate for the larvae, which eat living or dead tissue and physiological fluids. For older people, especially those with diabetes, immunosuppression, and peripheral vascular disorders, myiasis is common and more likely to strike the feet. The illness may be entirely benign and not symptomatic, or in difficult situations, it may result in the patient's death. In this case, we saw myiasis in the left tibial osteosarcoma with no comorbidities.

## Case presentation

**Figure 1:**
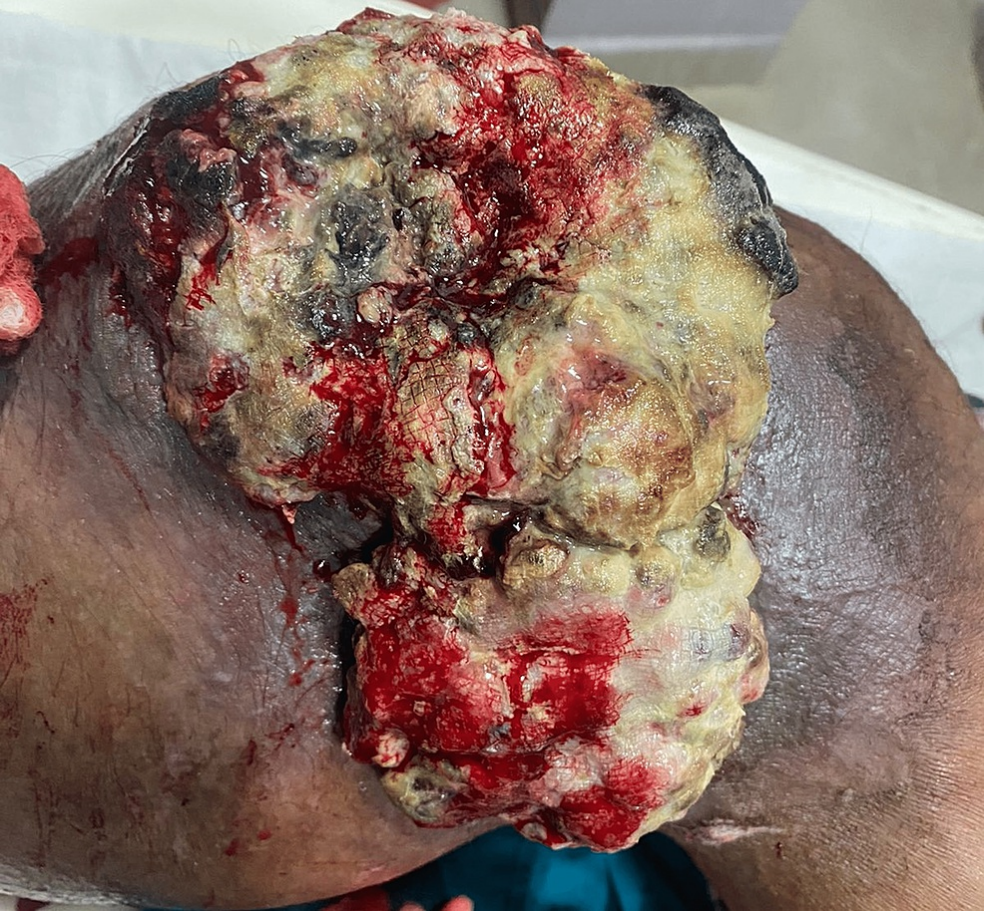
Fungating and cauliflower-shaped ulcers with everted edges and dilated veins. Figure credits: Harshika Kaur Saluja (author).

**Figure 2:**
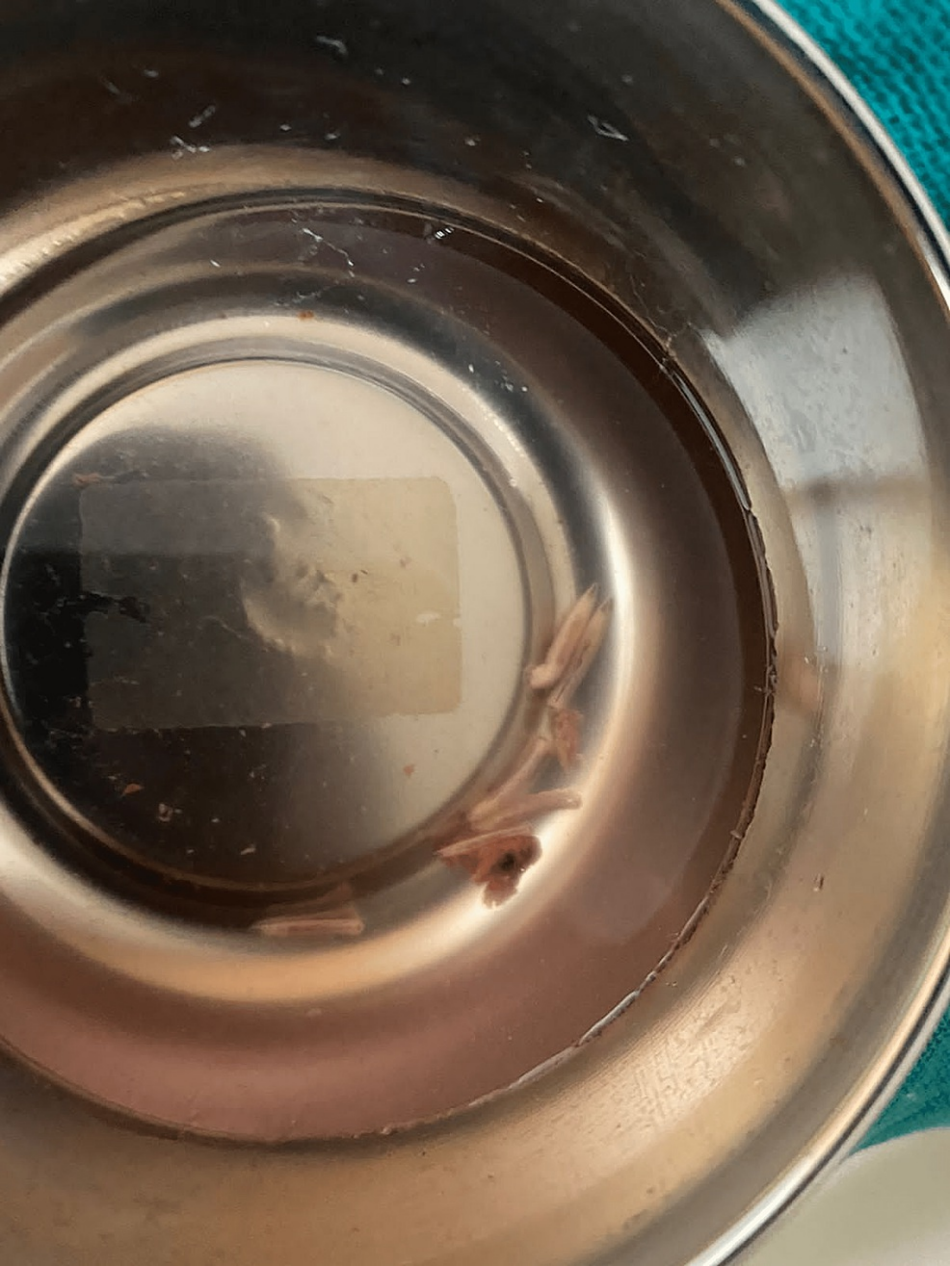
Image showing eight maggots isolated. Figure credits: Harshika Kaur Saluja (author).

A 45-year-old menopausal woman with unremarkable medical and surgical history and no comorbidities presented to our institute with pain in her left leg and tingling. She had been seeing a local doctor for the same issue after a diagnosis of osteosarcoma of proximal 1/3 diaphysis was made eight months ago. There was no prior trauma, night-time temperature rise, weight loss, or loss of appetite. Upon inspection, the proximal end of the left tibia and knee showed slight oedema. When touched, the swelling felt tender. There was no crepitus or abnormal movement. On examination, fungating and cauliflower-shaped ulcers with everted edges were present. It was foul-smelling, friable on touch, bleeding easily, and dilated veins were present (Figure 1). Paresthesia was not noted. A total of eight maggots (Figure 2) were isolated, and turpentine oil was applied hourly. Alkaline phosphatase (ALP) and lactate dehydrogenase (LDH) levels in her blood were within normal ranges.

**Figure 3:**
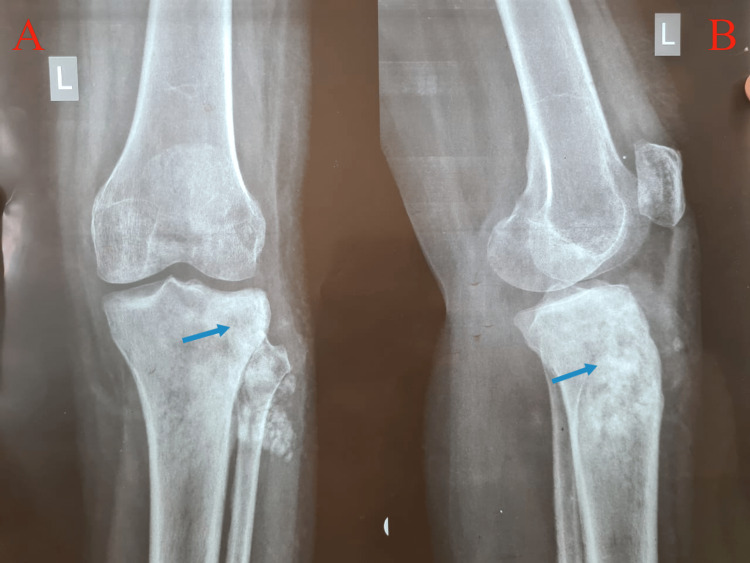
X-ray of the knee at the time of presentation. (A) Anteroposterior and (B) lateral view showing osteolytic lesion with periosteal reaction and tumour surrounding in the proximal 1/3 of the shaft of the tibia (blue arrows). Figure credits: Harshika Kaur Saluja (author).

**Figure 4:**
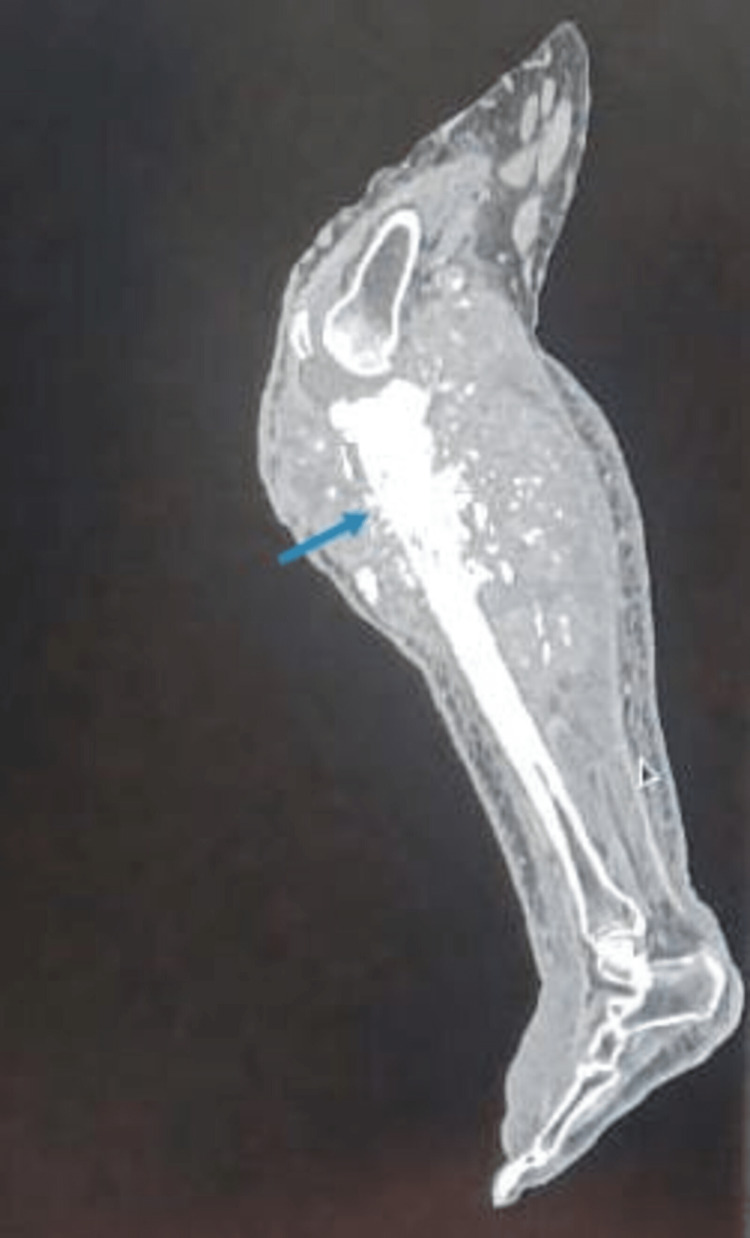
CT scan image of the tumour showing the involved tibia (blue arrow). Figure credits: Harshika Kaur Saluja (author).

**Figure 5:**
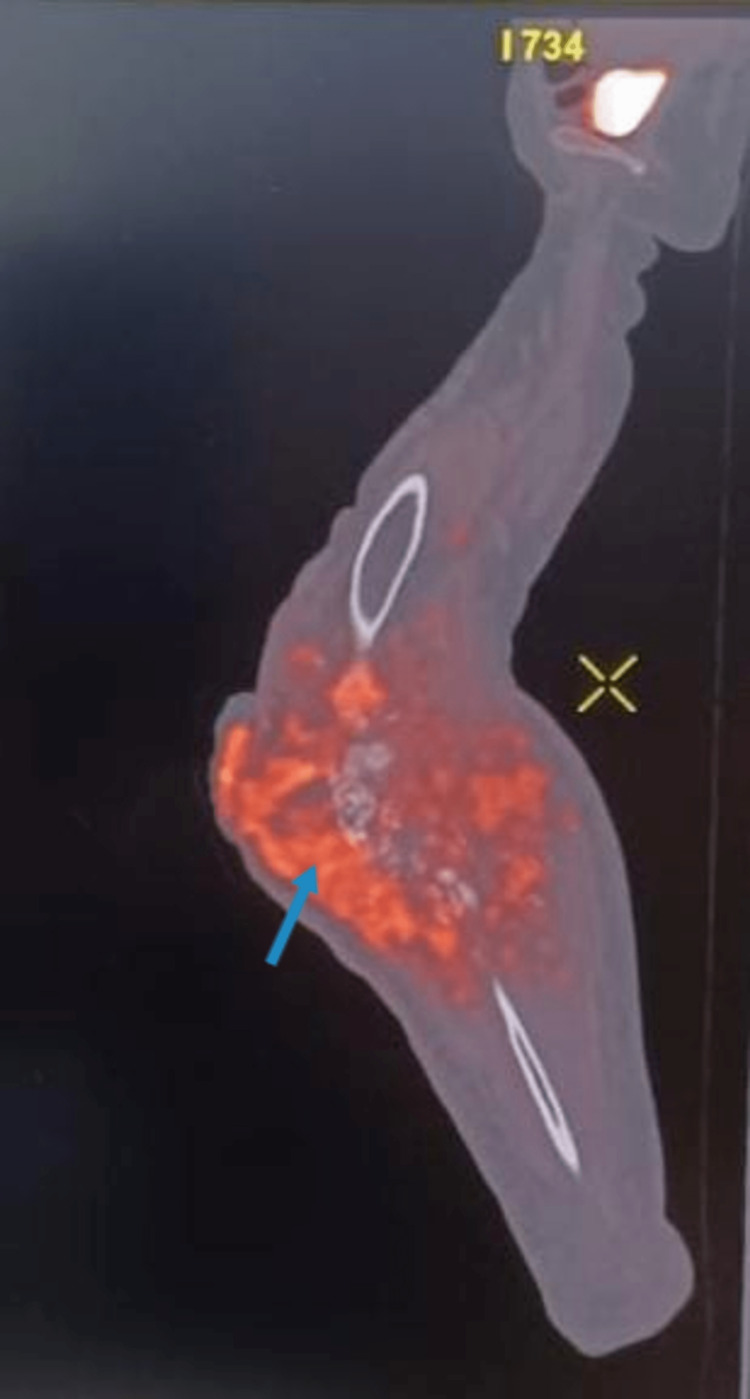
Positron emission tomography-computed tomography showing enhanced tumour lesions with involved soft tissue around proximal tibia and fibula (blue arrow). Figure credits: Harshika Kaur Saluja (author).

In the radiographs, the affected limb’s X-ray revealed an osteolytic lesion with periosteal reaction and tumour surrounding the proximal 1/3 of the shaft of the tibia (Figure 3). Computed tomography (CT) scan showed involvement of proximal diaphysis of the tibia (Figure 4). Magnetic resonance imaging (MRI) showed extensive destruction of the cortex of the proximal tibia and fibula, along with soft tissue involvement. Positron emission tomography-CT (PET-CT) showed enhanced tumour lesions involving soft tissue around the proximal tibia and fibula (Figure 5).

Sheets of cancerous cells could be seen in tissue slices. These cells had hyperchromatic nuclei, scanty cytoplasm, and round to oval or spindle-shaped bodies. They also had an increased nucleus-to-cytoplasmic (N:C) ratio. Osteosarcoma was determined to be the cause of the lesion based on its malignant character, location, and results from MRI and other radiographs. Accordingly, neoadjuvant chemotherapy was started with an injection of adriamycin 40 mg from day one to day three and an injection of cisplatin 50 mg on day two and day three. Additionally, surgical debridement of necrotic tissue was performed along with the mechanical removal of larvae using turpentine oil. All of the maggots were eliminated during the subsequent five days of wound debridement. Systemic antibiotics, metronidazole, and augmentin (amoxicillin + clavulanic acid) were given.

## Discussion

In the United States, there are 400 cases of osteosarcoma per year (4.8 cases per million people under the age of 20 years). Patients whose disease was identified between 1974 and 1994 had a five-year overall survival rate of 63% (59% for men and 70% for women). Compared to Caucasians, African Americans have a somewhat greater incidence of the disease. Males are slightly more likely to develop this disease than females. The incidence for males is 5.2 cases/million/year. Children under the age of five are less likely to develop osteosarcoma. Approximately 0.5 cases per million persons are affected annually [2,6]. The incidence increases gradually with age; during adolescence, a somewhat abrupt rise coexists with the growth surge [2]. According to a study in India, osteosarcoma accounts for the greatest percentage (36%) of instances among the many primary bone cancers, followed by chondrosarcoma, osteoclastoma, and Ewing's sarcoma [7]. Patients with osteosarcoma above 40 years of age in an Asian population had an incidence of 13-30% [8]. Low disease prevalence has been reported in Asia and the Middle East [9]. Pain is the most common initial symptom of osteosarcoma, especially when moving around. Patients may express discomfort related to arthritis, growing pains, or sprains. Even though pathologic fractures are uncommon, the patient frequently has a history of trauma. The telangiectatic type of osteosarcoma is an exception, as it often co-occurs with pathologic fractures [10]. A limp can develop if lower extremity pain is present. Systemic symptoms like fever or night sweats are pretty uncommon [2]. Respiratory symptoms from lung metastases are unusual and, if present, typically signal substantial lung involvement. Since metastases to other sites are comparatively uncommon, additional symptoms are exceptional. Metastases, which mostly affect the lungs but can also affect other bones, are only evident in 15-25% of patients at the time of presentation [2]. Multiple skeletal lesions at the time of diagnosis are typical for multifocal sclerosing osteosarcoma [10-12]. The primary tumour site corresponds with its physical findings such as soft tissue mass, decreased range of motion, lymphadenopathy, and respiratory symptoms. There is no recognised aetiology for osteosarcoma. However, some risk factors have been described. The only known environmental risk factor is radiation exposure [2].

Radiographic features alone cannot be used for diagnosis. Solely osteolytic lesions make up about 30% of all osteosarcomatous lesions, and purely osteoblastic lesions make up about 45% of cases. The recognisable Codman triangle may be seen when the periosteum is elevated [2,3]. About 60% of patients who have periosteal tumour extension may exhibit a so-called sunburst look. The whole bone and nearby joints should be scanned to check for skip lesions and joint involvement. Osteosarcomas with telangiectasia are frequently cystic and might be misinterpreted as aneurysmal bone cysts. To check for pulmonary metastases, chest radiographs with posteroanterior (PA) and lateral views should be taken. For surgical planning, a CT scan of the primary lesion is essential for determining the location and size of the tumour. Chest CT is more sensitive than conventional radiography for determining lung spread. To eliminate the uncertainty resulting from post-anaesthesia atelectasis, the chest CT should ideally be performed, and then the biopsy [11,12]. The degree of the disease, as determined at the time of definitive surgery, best correlates with the results of an MRI. To rule out slipping lesions, an MRI should additionally incorporate joint-to-joint imaging. It is crucial to evaluate radionuclide bone scan results using technetium-99m-methylene diphosphonate (Tc-99 MDP) for the existence of metastatic or multifocal illness. Then, a CT or MRI should be used to image the abnormal areas [10,11]. The two most crucial components of the tumour's histological evaluation are first, by looking at the biopsy material, the type of the tumour can be determined. Second, assessing the tissue removed after chemotherapy is the only way to determine the treatment's effectiveness [2].

In childhood and adolescence, the conventional form is most prevalent. The osteoblastic, chondroblastic, and fibroblastic kinds have been used to further categorise this type, despite the clinical indistinguishability of the subtypes. It is a grade 3 or 4 tumour. The fibroblastic variant of chondroblastic osteosarcoma features a spindle cell stroma with focal osteoid and produces cartilage. Staging tumours aims to identify different risk categories [2].

Before the advent of chemotherapy in the 1970s, surgical resection, typically amputation, was the mainstay of osteosarcoma treatment. Despite having such strong local disease management, more than 80% of patients went on to develop recurrent illness, which usually shows up as lung metastases. Most patients had micrometastatic disease at diagnosis, inferred from the high recurrence rate. To effectively treat patients with osteosarcoma, systemic adjuvant chemotherapy is crucial. Neoadjuvant chemotherapy facilitates surgical tumour removal once the tumour has decreased, and it also gives oncologists an important risk parameter [13-15].

The prognosis is better for patients whose tumours react favourably to neoadjuvant chemotherapy (>95% cancer cell death or necrosis) than for those whose tumours do not. Surgery is the only treatment available for osteosarcomas because radiation is ineffective in removing the tumour (i.e., local control) [12]. Doxorubicin, cisplatin, and high-dose methotrexate are the chemotherapy agents that are most effective against osteosarcoma. Open biopsy is advised since it reduces sampling error and gives considerable tissue for biological research. The primary goal of definitive resection is patient survival. The patient often prefers limb salvage reconstruction to amputation. Still, recent research has shown that amputated patients can maintain a long-term life quality comparable to individuals with limb salvage.

## Conclusions

In this case report, we presented a case of osteosarcoma of the proximal 1/3 left tibia in the diaphyseal region, which is extremely rare, as osteosarcoma preferably grows near the metaphysis. The diagnosis was confirmed with MRI and other radiographs. The approach towards the treatment and an attempt to improve the prognosis was made by starting neoadjuvant chemotherapy. The patient came for a follow-up after the chemotherapy, but with such extent of the mass, the final treatment planned was amputation.
